# Comparison of the Effects on Rib Fracture between the Traditional Japanese Medicine Jidabokuippo and Nonsteroidal Anti-Inflammatory Drugs: A Randomized Controlled Trial

**DOI:** 10.1155/2012/837958

**Published:** 2012-07-24

**Authors:** Hajime Nakae, Aya Yokoi, Hiroyuki Kodama, Akira Horikawa

**Affiliations:** ^1^Department of Emergency and Critical Care Medicine, Akita University Graduate School of Medicine, 1-1-1 Hondo, Akita 010-8543, Japan; ^2^Department of Traditional Japanese Medicine, Akita University Hospital, 1-1-1 Hondo, Akita 010-8543, Japan; ^3^Division of Orthopedics, Minamiakita Orthopedic Clinic, 96-2 Kaidoshita, Syowaokubo, Katagami 018-1401, Japan; ^4^Division of Orthopedics, Yuzawa Clinic, 3-22 Satake Cho, Yuzawa 012-0824, Japan

## Abstract

Jidabokuippo is a traditional Japanese medicine used for contusion-induced swelling and pain. This open multicenter randomized study was designed to compare the efficacies of jidabokuippo and nonsteroidal anti-inflammatory drugs (NSAIDs) in patients with rib fracture by analyzing the treatment duration. Our study involved 170 rib fracture patients capable of oral ingestion divided randomly into 2 groups: the jidabokuippo and NSAID groups. We compared the duration of treatment and healthcare expenditure between these 2 groups. Medication was continued in both groups until the visual analogue scale score decreased to less than 50% of the pretreatment score. We excluded the patients in whom medication was prematurely discontinued. We analyzed 81 patients belonging to the jidabokuippo and NSAIDs groups. No significant intergroup differences were observed in age, gender, severity (injury severity score), and presence/absence of underlying disease. The treatment duration was significantly shorter in the jidabokuippo group than in the NSAIDs group (*P* = 0.0003). Healthcare expenditure was significantly lower in the jidabokuippo group than in the NSAIDs group (*P* < 0.0001). Our results suggest that compared to NSAIDs, jidabokuippo can shorten the duration of treatment in patients with rib fracture and is a promising analgesic agent based on the medical economic viewpoint.

## 1. Introduction

Jidabokuippo is a herbal mixture created in Japan for contusion-induced swelling and pain; jidabokuippo is composed of the herbs Nuphar Rhizome (*Nupharis Rhizoma*), Quercus Bark (*Quercus Cortex*), Cnidium Rhizome (*Cnidii Rhizoma*), Cinnamon Bark (*Cinnamomi Cortex*), Clove Floral Bud (*Caryophylli Flos*), Rhubarb Rhizome (*Rhei Rhizoma*), and Glycyrrhiza Root (*Glycyrrhizae Radix*) [1]. The herbs composing jidabokuippo have antioxidant effects [2–11]. In addition, we have reported previously that jidabokuippo has antioxidant activity [12, 13]. Swelling observed after trauma is caused by elevated vascular permeability based on the synthesis of chemical mediators, including free radicals. Therefore, antioxidant activity is also likely to be involved in the alleviation of swelling. Usually, nonsteroidal anti-inflammatory drugs (NSAIDs) are used for the treatment of pain associated with trauma. However, NSAIDs sometimes induce gastrointestinal symptoms even in patients concomitantly treated with antiulcer drugs and thus hinder the continuation of NSAID treatment [14–17]. Pain responses vary greatly among individuals, and the visual analogue scale (VAS) is often used for evaluating the responses of pain to treatment. However, the evaluation based on VAS score lacks objectivity because it relies on patient self-reports. Patients tend to discontinue consumption of an analgesic if pain is alleviated to an endurable level, which affects the duration of treatment and consequently reflects responses to treatment. Thus, in this study we compared the efficacies of jidabokuippo and NSAIDs in patients with rib fractures by analyzing the duration of treatment.

## 2. Methods

We performed a randomized, multicenter, prospective, nonblinded, clinical trial in which we compared the efficacy of jidabokuippo and NSAIDs in 170 patients with rib fracture at Akita University Hospital, Minamiakita Orthopedic Clinic, and Yuzawa Clinic between January 1, 2009, and May 31, 2011. Informed consent was obtained from all of the patients and their families involved at the time of their enrollment. The study was performed with the approval of the ethic committee of the Akita University Hospital and was performed in accordance with the guidelines of good clinical practice.

Rib fractures were diagnosed by radiographic examination (chest radiographs and a computed tomography (CT) scan of the chest).

Patients incapable of oral ingestion because of multiple injuries and those who visited the hospital after 4 days or more after the injury were excluded from this study. Pregnant women and children younger than 15 years were excluded as well.

**Figure 1:**
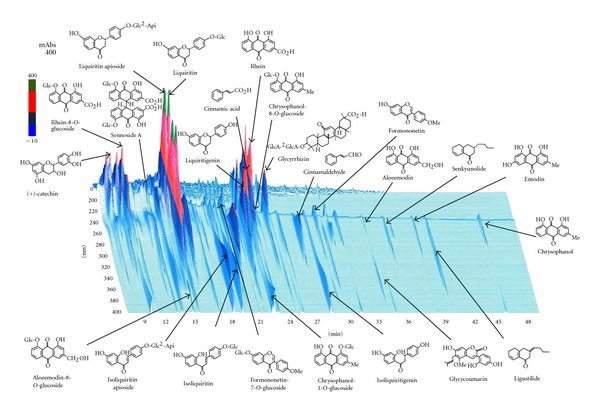
Three-dimensional high-performance liquid chromatography (HPLC) profile of jidabokuippo.

Patients were randomized into one of the 2 treatment groups using the envelope method before administration of medication. The patients allocated to the jidabokuippo group took granular type extract (TJ-89; Tsumura & Co., Tokyo, Japan), and concomitant consumption of only herbal medicines was permitted. The jidabokuippo consists of 7 herbs shown in Table 1. The three-dimensional high-performance liquid chromatography (HPLC) chart of the methanol solution of jidabokuippo is shown in Figure 1. Jidabokuippo preparation was obtained with 20 mL of methanol under ultrasonication for 30 min. The solution was filtered and submitted to HPLC analysis [18]. HPLC equipment was controlled with an HPLC pump (LC-10AD; Shimazu, Kyoto, Japan) using a TSK-GEL, ODS-80TS column (4.6*φ* × 250 nm), and elution was performed using solvents (A) 0.05 M ammonium acetate (AcONH_4_; pH, 3.6) and (B) acetonitrile (CH_3_CN). A linear gradient of 100% A and 0% B changing over 60 min to 0% A and 100% B was used. The flow rate was controlled with LC-10AD at 1.0 mL/min. The eluate from the column was monitored, and the three-dimensional data were processed with a diode array detector (SPD-M10A; Shimadzu, Kyoto, Japan).

For patients allocated to the NSAIDs group, the type of NSAID used and the concomitant use of other drugs were decided by the attending physicians. The decision to discontinue treatment (for reasons such as poor responses, and adverse reactions) was assigned to the attending physician. Treatment was continued until the VAS score decreased to less than 50% of the initial score, and patients who prematurely discontinued were excluded from analysis.

No limit was set to the concomitant use of cardiovascular drugs (antihypertensive, antiarrhythmic and antihyperlipidemic drugs), antiplatelet drugs, neuropsychiatric drugs (antianxiety and antiepilepsy drugs), gastrointestinal drugs, hormone preparations, bone metabolism-improving agents, and so forth, in patients who prescribed these drugs before the start of the study.

During the course of rib fracture treatment, many patients stop visiting the facility if pain is relieved even before complete bone fusion is achieved. In addition, many patients with rib fractures see no necessity to take an analgesic despite the presence of a slight pain during body motions or coughing. Considering these factors, we analyzed the duration of treatment as index. In addition, we determined the medical expense to evaluate its superiority from the viewpoint of medical economics.

On the basis of a previous prospective pilot study (*n* = 74, unpublished data), we set up the following hypothesis: the minimal clinical requirement to endorse a significant difference is a 5-day reduction in the treatment duration in the jidabokuippo group compared to that in NSAIDs group when the standard deviation in the treatment duration among patients with rib fracture is 11 days. Under this assumption, we required 77 subjects for each group to achieve a detection power over 80%. Assuming that about 4% of the subjects would be excluded from analysis for reasons such as adverse events [14–16], the number of subjects required is 80.2/group. Thus, we set the number of subjects at 85/group.

Each parameter was expressed as the median (minimum-maximum) value. The Mann-Whitney *U* test and Wilcoxon signed-ranks test were used for comparisons between the 2 groups. The Kruskal-Wallis rank test was used for comparisons among the 3 groups. In addition, Fisher's exact test was employed. *P* < 0.05 was regarded as statistically significant.

We are planning perprotocol analyses after study completion.

## 3. Results

**Figure 2:**
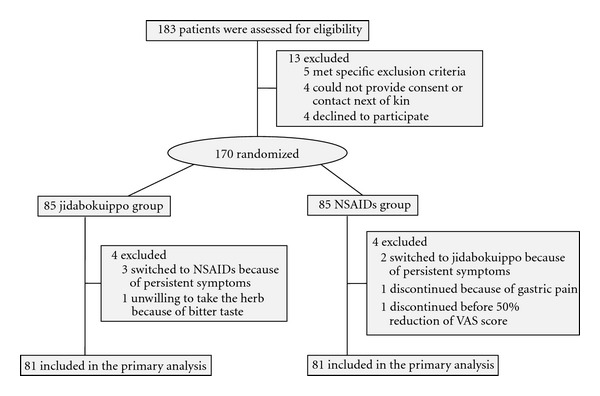
Study flow diagram.

Of the 183 patients who underwent screening for eligibility, 170 underwent randomization (Figure 2). Of the 85 patients allocated to the jidabokuippo group, 4 were excluded from analysis. These 4 patients included 3 in whom jidabokuippo was switched to NSAIDs because of lack in symptom alleviation and 1 in whom the herb complex was discontinued at the patient's discretion because of unacceptable taste. Of the 85 patients in the NSAIDs group, 4 were excluded from analysis. Among them, 2 switched to jidabokuippo because of lack of symptom alleviation, 1 discontinued because of gastrointestinal symptoms, and 1 discontinued before the VAS score reduced to less than 50% of the initial score. The incidence of adverse events (gastrointestinal symptoms, etc.) did not differ significantly between the 2 groups (jidabokuippo group: 0% [0/85] versus NSAIDs group: 5.9% [5/85], *P* = 0.0588).

We analyzed 81 patients of jidabokuippo and 81 patients of NSAIDs groups (Figure 2). In the jidabokuippo group, 74 patients did not take combined therapy and 7 received combined therapy with Kampo diagnosis. The Kampo medicines concomitantly used in the jidabokuippo group were Goshajinkigan (4 patients), Shakuyakukanzoto (2), Hachimijiogan (1), and Aconite Tuber (1). One patient received 3 herbal medicines. Drugs used in the NSAIDs group were loxoprofen (34 patients), diclofenac sodium (22), lornoxicam (14), etodolac (8), meloxicam (6), celecoxib (4), and naproxen (1). Six patients received more than 2 kinds of NSAIDs. All patients in the NSAIDs group received drugs for gastritis/gastric ulcer, but not proton pump inhibitors (PPIs). No patients in both groups did not take acetaminophen, narcotics, pain catheters, epidurals, or rib fixation and had previously received jidabokuippo. Accordingly we could get the good results.

The data on background variables is summarized in Table 2. Median age did not differ significantly between the 2 groups (jidabokuippo group: 60 years [19–90 years] versus NSAIDs group: 66 years [23–90 years], *P* = 0.2553). The men-to-women ratio did not differ between the 2 groups (jidabokuippo group: 35 : 46 versus NSAIDs group: 37 : 44, *P* = 0.8744). The ratio of the number of rib fractures did not differ significantly between the 2 groups (jidabokuippo group: 74 : 7 versus NSAIDs group: 75 : 6, *P* > 0.9999). While 2 patients in the jidabokuippo group had bilateral fractures, none in the NSAIDs group had bilateral fractures. No patients in both groups had flail chest. The ratio of the site of rib fractures did not differ significantly between the 2 groups (jidabokuippo group, upper [1st–4th]: 5 patients; middle [5th–8th]: 34 patients; lower [9th–12th]: 46 patients versus NSAIDs group, upper: 5 patients; middle: 43 patients; lower: 37 patients, *P* = 0.3620). Median injury severity score (ISS) did not differ significantly between the 2 groups (jidabokuippo group: 1 [1–10] versus NSAIDs group: 1 [1–13], *P* = 0.8050). Median chest abbreviated injury scale score did not differ significantly between the 2 groups (jidabokuippo group: 1 [1–3] versus NSAIDs group: 1 [1–3], *P* = 0.7390). The percentage of accompanying injuries did not differ significantly between the 2 groups (jidabokuippo group: 4 [4.9%] patients [whiplash injury: 3 patients; kidney contusion: 1 patient] versus NSAIDs group: 6 [7.4%] patients [hemothorax: 2 patients; pneumohemothorax: 1 patient; pneumothorax: 1 patient; clavicular fracture: 2 patients; whiplash injury: 1 patient], *P* = 0.7441).

The percentage of patients with comorbidities did not differ significantly between the 2 groups (jidabokuippo group: 19 [23.5%] patients versus NSAIDs group: 18 [22.2%] patients, *P* > 0.9999). The comorbidities are shown in Table 3. No significant difference was observed in each disease between the 2 groups.

**Figure 3:**
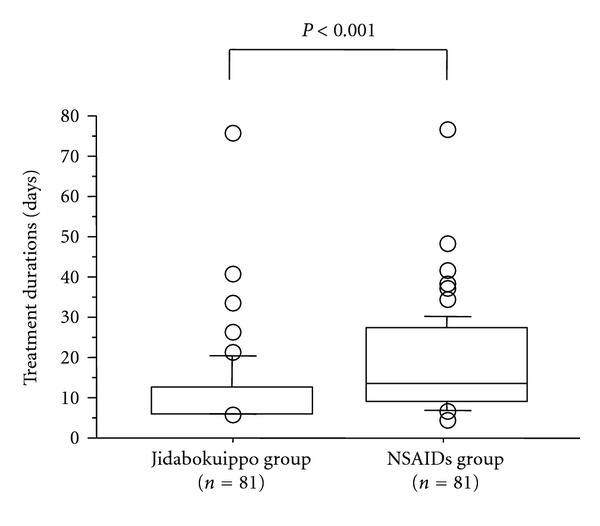
Comparison of treatment durations between the jidabokuippo and the NSAIDs groups. Median treatment duration was significantly lower in the jidabokuippo group than in the NSAIDs group (*P* < 0.001).

Comparison of the durations of treatment between the 2 groups is shown in Figure 3. Median duration of treatment was significantly lower in the jidabokuippo group (7 days [7–77 days]) than in the NSAIDs group (14 days [5–77 days], *P* = 0.0003).

Median expenditure for medication was significantly lower in the jidabokuippo group (509.3 Yen [339.5–5601.8 Yen] [6.29 US dollars, 4.20–69.24 US dollars]) than in the NSAIDs group (1581.3 Yen [468.3–10256.4 Yen] [19.54 US dollars, 5.79–126.77 US dollars], *P* < 0.0001).

The subgroup analysis between the 2 groups based on durations of treatment is summarized in Table 4. Median duration of treatment in men did not differ significantly between the 2 groups (*P* = 0.3783). Median treatment duration in women was significantly lower in the jidabokuippo group than that in the NSAIDs group (*P* < 0.0001). No significant differences were observed between men and women in the jidabokuippo groups (*P* = 0.3498). Median duration of treatment in men was significantly lower than that in women in the NSAIDs group (*P* = 0.0389). Median duration of treatment in single fracture was significantly lower in the jidabokuippo group than in the NSAIDs group (*P* < 0.0001). Median duration of treatment in multiple fractures did not differ significantly between the 2 groups (*P* = 0.4340). No significant differences were observed between single and multiple fractures in both groups (jidabokuippo group, *P* = 0.0658; NSAIDs group, *P* = 0.2245). Median duration of treatment in patients with upper rib fractures did not differ significantly between the 2 groups (*P* = 0.6664). Median duration of treatment in patients with middle rib fractures was significantly lower in the jidabokuippo group than in the NSAIDs group (*P* = 0.0020). Median duration of treatment in patients with lower rib fractures did not differ significantly between the 2 groups (*P* = 0.0934). No significant differences were observed between the sites of rib fractures in both groups (jidabokuippo group, *P* = 0.9488; NSAIDs group, *P* = 0.5869). Median duration of treatment in the group with accompanying injuries did not differ significantly between the 2 groups (*P* = 0.1224). Median duration of treatment in the group without accompanying injuries was significantly lower in the jidabokuippo group than in the NSAIDs group (*P* = 0.0006). No significant differences were observed between accompanying injuries in both groups (jidabokuippo group, *P* = 0.0833; NSAIDs group, *P* = 0.5072). Median duration of treatment in the group with comorbidities did not differ significantly between the 2 groups (*P* = 0.1130). Median duration of treatment in the group without comorbidities was significantly lower in the jidabokuippo group than in the NSAIDs group (*P* = 0.0007). No significant differences were observed between comorbidities in both groups (jidabokuippo group, *P* = 0.1533; NSAIDs group, *P* = 0.8945).

## 4. Discussion

The actions of the herbs comprising jidabokuippo are as follows: Nuphar Rhizome absorbs internal hemorrhage and repairs tissue; Quercus Bark has analgesic, detoxifying, anti-inflammatory, and hemostatic actions; Cnidium Rhizome has anti-inflammatory and analgesic actions; Clove Floral Bud and Rhubarb Rhizome improve microcirculation. Therefore, jidabokuippo is used to treat swelling and pain associated with trauma [1].

Plants are known to contain various antioxidants that protect organisms from injury caused by ultraviolet radiation and so forth [19–21]. Yamane et al. evaluated the radical-scavenging potentials of 7 herbs (Rhubarb Rhizome, Gambir [*Uncaria gambir*], Clove Floral Bud, Peony Root [*Paeoniae Radix*], Glycyrrhiza Root, Chuling [*Polyporus*], and Peach Kernel [*Presicae Semen*]) and reported the scavenging potential of diphenylpicrylhydrazyl (DPPH) was the highest in Rhubarb Rhizome followed by Clove Floral Bud [2]. Tani et al. suggested polyphenol to be closely involved in antioxidant effects on the basis of a positive correlation between polyphenol content and DPPH radical-scavenging potential of herbs [3]. They investigated 25 herbs. The polyphenol content was the highest in Rhubarb Rhizome, followed by Quercus Bark, Nuphar Rhizome, Glycyrrhiza Root, Clove Floral Bud, and Cinnamon Bark in this order. The DPPH radical-scavenging potential was high in Rhubarb Rhizome, followed by Quercus Bark, Nuphar Rhizome, Clove Floral Bud, and Cinnamon Bark in this order. In addition, Cnidium Rhizome has anti-inflammatory and antioxidant actions. A study designed to evaluate the effect of herb extracts in suppressing reactive oxygen formation in human neutrophils showed existence of suppressive action in Cnidium Rhizome [4]. In addition, this herb protects organisms from radiation-induced damage [5, 6], and also protects from edema [7]. Cinnamon Bark suppresses formation of reactive oxygen in aqueous extracts [8], inhibits O^2−^ formation in macrophages [9], and protects from radiation disorder [5]. Rhubarb Rhizome including anthraquinones suppresses formation of lipid peroxide in human neutrophils [10], condensed tannin has radical scavenging activity [11], and so forth. Glycyrrhiza Root has anti-inflammatory and edema-suppressing activities [22, 23]. In addition, Glycyrrhiza Root protects organisms from radiation [5]. Thus, jidabokuippo includes herbs with much antioxidant effects, and these herbs may play synergistically in antioxidant effects.

During the course of rib fracture treatment, many patients stop visiting the medical facility if pain is relieved even before complete bone fusion. In addition, many patients with rib fractures do not take analgesics despite slight persistent pain in body motions or coughing. Because evaluation with VAS is apt to result in large interindividual variance, we evaluated the duration of analgesic medication as an indicator of analgesic effects in the present study. The duration of treatment was significantly shorter in the jidabokuippo group than in the NSAIDs group. Crossover did not differ significantly between the jidabokuippo (3.75%) and the NSAIDs (2.5%) groups, which suggested that jidabokuippo is not inferior to NSAIDs in the potency of analgesic activity. However, it remains unclear whether the jidabokuippo stimulates healing or NSAIDs require a longer time to heal than in the natural course because NSAIDs suppress prostaglandins simultaneously with the suppression of wound-healing cytokines despite their analgesic activity. These issues remain to be solved in the future.

NSAIDs are often used for the pain associated with trauma. However, intake of NSAIDs often induces gastrointestinal symptoms [14, 15]. In addition, the use of selective cyclooxygenase-2 inhibitors has a risk of ischemic heart disease [24], and medical doctors sometimes hesitate to use them in patients with a history of cardiovascular disease. In the recent years, PPIs began to be used for the prevention of NSAID-induced ulcers. However, PPIs have problems in medical economics, fracture, community-acquired pneumonia, watery stools, and so forth, [15, 25]. In the present study, no adverse events occurred in the jidabokuippo group. On the other hand, 2 (2.5%) patients in the NSAIDs group developed gastrointestinal symptoms despite intake of drugs for gastritis/gastric ulcers. Continuation of NSAID treatment was not possible in the case of these 2 patients. Although the incidence of adverse events did not differ significantly between the 2 groups, our results suggest that jidabokuippo can be used more safely than NSAIDs. However, 1 patient in the jidabokuippo group discontinued it because of bitter taste (based on a problem with dosage). Herbal medicines are usually dissolved in warm water and therefore are more difficult to take than a tablet form. Accordingly, herbal medicines in tablets or capsules would be desirable. 

Cost reduction is an important issue in healthcare. In general, herbal medicines are cheap. Our study showed that medication expense was significantly lower in the jidabokuippo group than in the NSAIDs group; partially based on the shortage of the duration of treatment. Our results suggested that jidabokuippo is superior to NSAIDs in terms of analgesic effects and the economic viewpoint. We used different kinds of NSAIDs in this study. A double-blind study with one NSAID as a control arm in a large number of patients with trauma will be necessary to confirm the excellent effectiveness of jidabokuippo in future. We are planning to compare the effects of jidabokuippo in patients with various types of trauma.

## 5. Conclusions

Duration of treatment was significantly shorter in the jidabokuippo than in the NSAIDs group. This result suggests that jidabokuippo can shorten the duration of treatment in patients with rib fracture compared with NSAIDs and that the former is promising as an excellent analgesic based on the medical economic viewpoint.

## Figures and Tables

**Table 1:** The 7 herbs that compose jidabokuippo and their dry weight/daily dose.

Latin names	Crude drugs	Weight (g)
*Cinnamomi Cortex*	Cinnamon Bark	3.0
*Cnidii Rhizoma*	Cnidium Rhizome	3.0
*Nupharis Rhizoma*	Nuphar Rhizome (Yellow Pond Lily)	3.0
*Quercus Cortex*	Quercus Bark	3.0
*Glycyrrhizae Radix*	Glycyrrhiza Root, Glycyrrhiza	1.5
*Rhei Rhizoma*	Rhubarb Rhizome, Rhubarb (Rhubarb)	1.0
*Caryophylli Flos*	Clove Floral Bud, Clove	1.0

**Table 2:** Patient demographics and clinical characteristics.

	Jidabokuippo group	NSAIDs group	*P* value
Age (years)	60 (16–90)	66 (23–90)	0.2553
Gender (male : female)	35 : 46	37 : 44	0.8744
The number of rib fractures (single : multiple)	74 : 7	75 : 6	>0.9999
	(bilateral, 2)	(bilateral, 0)	
Site of rib fractures	Upper (1st–4th) 5	Upper (1st–4th) 5	0.3620
	Middle (5th–8th) 34	Middle (5th–8th) 43	
	Lower (9th–12th) 46	Lower (9th–12th) 37	
Injury severity score	1 (1–10)	1 (1–13)	0.8050
Chest AIS score	1 (1–3)	1 (1–3)	0.7390
Accompanying injuries	4	6	0.7441
		Hemothorax 2	
		Pneumohemothorax 1	
	Whiplash injury 3	Pneumothorax 1	
	Kidney contusion 1	Clavicular fracture 2	
		Whiplash injury 1	
Comorbidities	19 (23.5%)	18 (22.2%)	>0.9999

ISS: injury severity score; AIS: abbreviated injury scale; NSAIDs: nonsteroidal anti-inflammatory drugs.

**Table 3:** Comorbidities.

Jidabokuippo group		NSAIDs group	
Hypertension	7	Hypertension	5
Diabetes mellitus	4	Osteoporosis	2
Osteoporosis	3	Diabetic mellitus	2
Hyperlipidemia	2	Bronchial asthma	2
Rheumatoid arthritis	1	Chronic bronchitis	1
Cerebral infarction	1	Rheumatoid arthritis	1
Myocardial infarction	1	Cerebral infarction	1
Atrial fibrillation	1	Intracerebral hemorrhage	1
Chronic bronchitis	1	Myocardial infarction	1
Chronic hepatitis	1	Atrial fibrillation	1
Chronic pancreatitis	1	Chronic hepatitis	1
Spondylolisthesis	1	Chronic renal failure	1
Sarcoidosis	1	Insomnia	1

NSAIDs: nonsteroidal anti-inflammatory drugs.

**Table 4:** Subgroup analysis based on treatment durations between the jidabokuippo and the nonsteroidal anti-inflammatory drug groups.

	Jidabokuippo group (number)	NSAIDs group (number)	*P* value
Gender			
Male	7 days (7–77 days) (35)	14 days (7–77 days) (37)	0.3783
Female	7 days (7–42 days) (46)	17 days (5–42 days) ^∗^(44)	<0.0001
The number of rib fractures			
Single	7 days (7–77 days) (74)	14 days (5–77 days) (74)	<0.0001
Multiple	14 days (7–23 days) (7)	13 days (7–28 days) (7)	0.4340
Site of rib fractures			
Upper	7 days (7–42 days) (5)	14 days (7–28 days) (5)	0.6664
Middle	10 days (7–23 days) (34)	14 days (5–49 days) (43)	0.0020
Lower	7 days (7–77 days) (46)	14 days (7–77 days) (37)	0.0934
Accompanying injuries			
(+)	7 days (7–14 days) (4)	14 days (7–28 days) (6)	0.1224
(−)	7 days (7–77 days) (77)	14 days (5–77 days) (75)	0.0006
Comorbidities			
(+)	14 days (7–28 days) (19)	14 days (7–42 days) (18)	0.1130
(−)	7 days (7–77 days) (62)	14 days (5–77 days) (63)	0.0007

^∗^ *P* < 0.05, male versus female; NSAIDs: nonsteroidal anti-inflammatory drugs.
